# An Anaerobic Trickle-Bed Reactor Filled with Siporax™ as a Novel Approach for Biomethanation of Hydrogen and Carbon Dioxide

**DOI:** 10.3390/bioengineering13040382

**Published:** 2026-03-26

**Authors:** Gert Hofstede, Arjan Kloekhorst, Janneke Krooneman, Kemal Koç, Kor Zwart, Folkert Faber, Jan-Peter Nap, Gert-Jan Euverink

**Affiliations:** 1 EnTranCe, Centre of Expertise Energy, Institute for Life Science & Technology, Hanze University of Applied Sciences, Zernikelaan 17, 9747 AA Groningen, The Netherlands; a.kloekhorst@pl.hanze.nl (A.K.);; 2 Engineering and Technology Institute Groningen (ENTEG), Products and Processes for Biotechnology, Faculty of Science and Engineering, University of Groningen, Nijenborgh 4, 9747 AG Groningen, The Netherlands; g.j.w.euverink@rug.nl; 3 Research Centre for Biobased Economy (RCBBE), Hanze University of Applied Sciences, Zernikeplein 11, 9747 AS Groningen, The Netherlands

**Keywords:** biogas upgrading, biomethanation, energy storage, hydrogen, methane formation rate, microbial resilience, renewable energy integration, trickle-bed reactor

## Abstract

To broaden the application of biomethanation for energy storage and renewable integration, this study investigates the performance of a trickle-bed reactor (TBR) for hydrogen (H_2_) utilisation in biogas upgrading, using both pure Carbon dioxide (CO_2_) and biogas-derived CO_2_ as substrates for methane (CH_4_) production. Renewable sources such as wind and solar are inherently variable, increasing the need for scalable storage solutions. Converting surplus electricity into H_2_ and CH_4_ via biological methanation offers an efficient and safer alternative to direct H_2_ storage. By reducing CO_2_ produced by biogas plants, methanogenic archaea produce CH_4_, enabling H_2_ valorisation and enhanced biogas yields. This study demonstrates that TBR technology can achieve CH_4_ formation rates up to 15 L-CH_4_/L-reactor/day under optimised conditions. Siporax carrier material supported dense biofilm formation and effective gas–liquid mass transfer, facilitating high conversion efficiency. The system showed operational robustness, with rapid recovery after prolonged idle periods and stable production rates of 10–12 L-CH_4_/L/day. Wastewater was used as a realistic medium to assess reactor performance under complex, variable conditions. Reactor design focused primarily on enhancing gas–liquid mass transfer and supporting sustained microbial activity through adequate nutrient supply, ensuring sufficient buffer capacity to maintain pH stability. These results demonstrate the potential of TBR-based systems for high-rate, stable biomethanation and highlight their applicability in future energy infrastructures for integrating H_2_ through decentralised biogas upgrading.

## 1. Introduction

The challenges of renewable energy sources like wind and solar highlight their output variability, driving increased focus on energy storage solutions. While batteries [1] and flywheels [2] are promising options, they are not yet cost-effective [3]. An alternative solution is to store electricity as chemical energy, specifically in the form of H_2_, CH_4_ via biomethanation, or NH_3_. However, NH_3_ storage poses significant safety challenges: NH_3_ is hazardous and should be handled with caution. H_2_ storage, particularly, is challenged by its low energy density, safety risks, and high transportation costs [4]. Biomethanation offers a promising solution by converting H_2_ and CO_2_ into CH_4_, which has a higher energy density than H_2_ and is easier to store and transport.

Biomethanation is a biological process that converts CO_2_ and H_2_ into CH_4_ through the activity of hydrogenotrophic methanogens according to the reaction CO_2_ + 4H_2_ → CH_4_ + 2H_2_O (ΔH = −165.0 kJ/mol) [5]. The CO_2_ in this process can be obtained from various sources, such as biogas [6]. With H_2_ sourced from renewable energy sources such as solar or wind, biomethanation can be particularly interesting for both renewable energy storage and biogas upgrading, as it converts the CO_2_ fraction of biogas (approximately 40%) into CH_4_ while utilising H_2_ generated from surplus renewable electricity.

Alternative biogas upgrading technologies are commercially available, but most focus exclusively on physical CO_2_ removal rather than CO_2_ conversion [7]. Established technologies, including absorption [8], adsorption [9], membrane separation [10], and cryogenic separation [11,12], effectively increase CH_4_ concentration and are well documented at an industrial scale. However, these approaches do not enhance the energetic content of biogas beyond concentration effects.

In contrast, biological methanation is fundamentally constrained by H_2_ mass transfer. Due to the low solubility of H_2_ in water, gas–liquid mass transfer rates are limited, thereby restricting H_2_ availability to hydrogenotrophic methanogens and reducing overall process efficiency [13].

Conventional reactor configurations used for biological methanation exhibit inherent limitations when applied to H_2_-driven CO_2_ conversion. In continuously stirred tank reactors (CSTRs), the low solubility of H_2_ in water results in severe gas–liquid mass transfer limitations, limiting H_2_ availability to hydrogenotrophic methanogens and consequently the achievable CH_4_ formation rates. Upflow anaerobic sludge blanket (UASB) reactors offer improved biomass retention; however, their applicability for ex situ biomethanation is constrained by limited control over gas distribution, challenges in handling high H_2_ gas flows, and the risk of gas channelling, which can compromise process stability [14]. Membrane-assisted systems have been proposed to enhance H_2_ delivery, yet these configurations introduce additional system complexity, increased capital and operational costs, and membrane fouling risks, which hinder large-scale implementation.

In contrast, trickle-bed reactors (TBRs) combine immobilised biomass with a high specific gas–liquid interfacial area, enabling efficient H_2_ transfer to methanogenic biofilms while maintaining operational stability. The fixed-bed configuration allows decoupling of hydraulic and gas residence times, facilitating independent optimisation of nutrient supply and gas conversion. This makes TBRs particularly suitable for high-rate ex situ biomethanation, where H_2_ mass transfer and microbial retention are critical performance determinants. Consequently, TBR technology represents a relevant reactor concept for achieving high CH_4_ formation rates under thermophilic biomethanation conditions.

In view of the mass transfer and gas-handling limitations associated with conventional reactor configurations, this study focuses on the application of a trickle-bed reactor (TBR) for ex situ biomethanation, which provides a high specific gas–liquid interfacial area and enables effective immobilisation of hydrogenotrophic methanogens [15]. The performance of a TBR can be evaluated using the Methane Formation Rate (MFR, m^3^ CH_4_/m^3^ reactor/day), a critical parameter for assessing the efficiency of biogas production systems [15].

Previous studies have reported that TBRs achieve a high specific CH_4_ production, reaching up to ~15 m^3^ per m^3^ of reactor volume per day, which is considerably higher than other configurations, such as CSTRs at ~3.7 m^3^/(m^3^·d) and up-flow reactors at ~0.25 m^3^/(m^3^·d) ([16,17,18]). Moreover, the biomethane produced meets the quality standards for direct injection into the gas grid, as specified in the European standard, which outlines the specifications for biomethane for injection in the natural gas network [19].

This study investigates the performance of a thermophilic trickle-bed reactor (TBR) for ex situ biomethanation of CO_2_ via hydrogenotrophic methanogenesis. The objectives are to (i) quantify CH_4_ formation rates and H_2_ conversion efficiency under controlled operating conditions, (ii) assess reactor stability and operational robustness, including restart after prolonged idle periods, (iii) evaluate reactor performance using both pure CO_2_ and biogas as feed gas, and (iv) examine the applicability of wastewater as a trickling medium. The study focuses on experimental reactor performance and operational behaviour; detailed techno-economic assessment and scale-up modelling are beyond the scope of this work.

## 2. Materials and Methods

### 2.1. Trickle-Bed Reactor Configuration and Operation

A laboratory-scale trickle-bed reactor (TBR) made of transparent PVC with a total working volume of 15 L was constructed. The reactor had a diameter-to-height ratio of 13.6:1, which falls within the range reported for trickle-bed reactors applied in biological gas conversion processes (1.3–33.3) ([20,21]). The reactor consisted of two double-walled sections, each with an internal volume of 7.5 L, and was operated at 55 °C under thermophilic conditions using an external circulating water bath. The reactor was filled with 15 L of cylindrical carrier material (Siporax^®^, nominal diameter 15 mm; recycled glass-based material; manufacturer-reported specific surface area 270 m^2^ L^−1^). The reactor was inoculated with anaerobic sludge obtained from an industrial anaerobic digestion facility (Paques, Balk, The Netherlands). During operation, the reactor was continuously trickled with sieved liquid derived from methanogenic granular sludge. The trickling liquid was recirculated at a flow rate of 25 L h^−1^ and supplemented once with 2 mL of a 100× concentrated anaerobic nutrient solution (Anaerobic POWERMIX^®^, Paques, The Netherlands).

**Figure 1:**
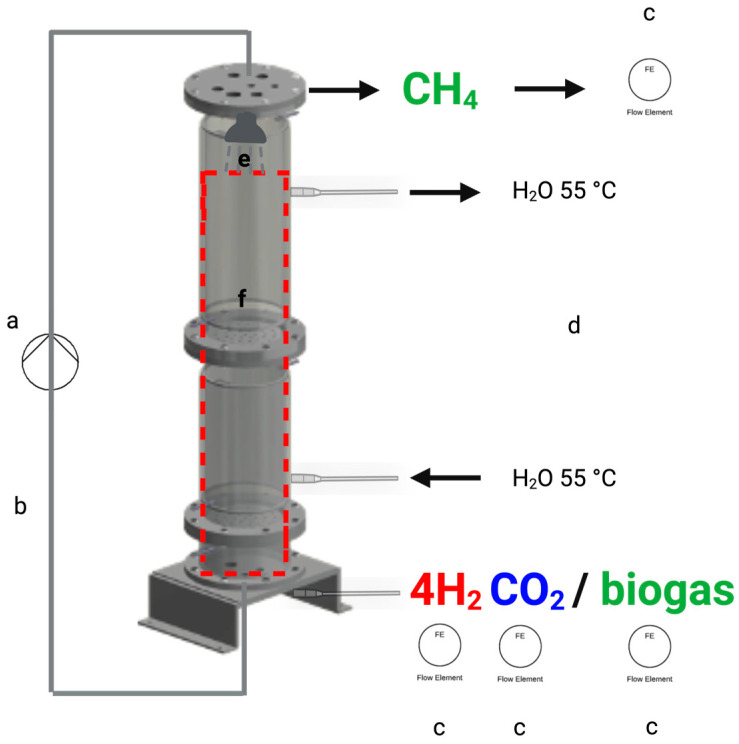
Configuration of a trickle-bed reactor for ex situ biomethanation: (**a**,**b**) represents counter-current flow, where gas and liquid phases move in opposite directions. Flowmeters are indicated by (**c**). Circulating water at 55 °C is shown in (**d**), while (**e**) denotes the shower distributor, and (**f**) refers to the carrier material, Siporax.

Following inoculation, the reactor was flushed with pure CO_2_ to establish anaerobic conditions and prevent local H_2_ accumulation. The reactor was initially operated with CO_2_ as the sole gas input for 24 h. Subsequently, H_2_ was gradually introduced to the gas feed. Gas flow rates and reactor off-gas production were continuously monitored using two mass flow metres (MV1 and MV2; Bronkhorst MV-102, The Netherlands). To determine CO_2_ production, the off-gas was passed through a 3 M NaOH solution to chemically remove CO_2_, after which the gas flow was measured again. CO_2_ in the off-gas was calculated from the difference between the measured inlet and outlet gas flows. Gas flow data were logged at one-minute intervals throughout the experiments. A schematic overview of the reactor configuration is provided in Figure 1.

### 2.2. Gas Composition Analysis

The composition of the reactor off-gas, including H_2_, CH_4_, and CO_2_, was analysed using a gas chromatograph (Shimadzu GC-2014, Shimadzu, 1, Nishinokyo Kuwabara-cho, Nakagyo-ku, Kyoto 604-8511, Japan) equipped with a packed molecular sieve column (Molesieve 5A; 1 m length, 1/8″ outer diameter, 2 mm inner diameter; Restek, PA, USA; catalogue no. 80440-800). Gas samples were collected from the reactor headspace using a gas-tight Hamilton syringe (50 µL; Hamilton, OH, USA), with an injected sample volume of 10 µL.

Chromatographic separation was performed with an initial column temperature of 110 °C, followed by a temperature ramp to 150 °C at 50 °C min^−1^, including an initial isothermal hold of 6 min and a final isothermal period of 3 min at 150 °C. The injector and thermal conductivity detector were both maintained at 150 °C. Nitrogen (N_2_) was used as the carrier gas at a flow rate of 8.0 mL min^−1^. Calibration gases containing known concentrations of H_2_, CH_4_, and CO_2_ were used to determine detector response factors for each gas component. Data acquisition and peak integration were performed using LabSolutions LC/GC software, version 5.111 (Shimadzu, 1, Nishinokyo Kuwabara-cho, Nakagyo-ku, Kyoto 604-8511, Japan).

## 3. Results

The performance of the trickle-bed reactor was evaluated based on the MFR and the composition of the reactor off-gas. Experiments were conducted using H_2_ and CO_2_ as substrates, as well as biogas containing both CH_4_ and CO_2_. Reactor performance was monitored over time by measuring gas flow rates, CH_4_ production, and H_2_ conversion under the applied operating conditions.

### 3.1. Characterisation of Siporax™ Carrier Material

The physical characteristics of Siporax™ (Hornbach, Groningerweg 45/2, 9738 AB Groningen) carrier material were quantified using a laboratory-scale column. Characterisation experiments were conducted in a reactor volume of 700 mL, which was adjusted to 1000 mL using a combination of whole and quarter Siporax™ rings. A fully packed column contained 273 g of whole rings or 387 g of quarter rings, corresponding to total specific surface areas of 270 m^2^ L^−1^ and 382 m^2^ L^−1^, respectively.

The pore volume of the carrier material was determined to be 185 mL L^−1^ for whole rings and 277 mL L^−1^ for quarter rings. The total volume occupied by the carrier material was measured by filling the column with water-saturated Siporax™ and subsequently adding water until the column was filled. The void volume was calculated from the additional volume of water required to fill the column and amounted to 314 mL L^−1^ for whole rings and 457 mL L^−1^ for quarter rings.

Based on the measured surface area and void volume, area-to-void-volume ratios of 859 m^2^ L^−1^ and 835 m^2^ L^−1^ were calculated for whole and quarter rings, respectively. The volume of open space (VOS), defined as the reactor volume minus the volume of the carrier material, was used to calculate the gas residence time (GRT). The gas residence time (GRT) was calculated as the ratio between the gas-filled volume (V_gas_) and the inlet gas flow rate (Q_gas_). At a gas flow rate of 10 mL min^−1^, the GRT was 31 min for whole rings and 46 min for quarter rings. When applying a H₂/CO₂ molar ratio of 3.7:1 (and assuming complete hydrogen conversion with H₂ as the limiting reactant), the gas volume decreases by a factor of 4.7, resulting in corresponding equivalent gas residence times of 147 min and 214 min for whole and quarter rings, respectively. Detailed calculations and underlying assumptions are provided in the Supporting Information (Section S1).

An overview of the measured and calculated characteristics of the Siporax™ carrier material is provided in Table 1.

### 3.2. Optimisation of CH_4_ Production in Laboratory-Scale TBRs

A series of laboratory-scale experiments was conducted to evaluate CH_4_ production performance under varying operational conditions. Experiments were performed in 1 L trickle-bed reactors filled with whole Siporax™ rings and inoculated with anaerobic sludge. The reactors were supplied with H_2_ and CO_2_, and CH_4_ production was monitored over time.

During the initial operation, CH_4_ production increased gradually and reached approximately 0.1 L CH_4_ L^−1^ d^−1^. After 25 days of operation, a maximum (MFR) of 4.3 L-CH_4_ L^−1^ d^−1^ was observed, after which the MFR stabilised at approximately 3 L-CH_4_ L^−1^ d^−1^. During this period, the circulating trickling liquid was refreshed weekly to maintain reactor operation.

Following adjustments to the reactor configuration and operating conditions, a substantial increase in CH_4_ production was observed. Under the modified conditions, the reactor reached a maximum MFR of 15 L-CH_4_ L^−1^ d^−1^ and subsequently stabilised at approximately 13 L-CH_4_ L^−1^ d^−1^. Similar conditions were applied in subsequent experiments.

Reactor performance was progressively optimised through three targeted modifications: (i) replacement of the original wastewater treatment inoculum with pre-enriched methanogenic granular sludge, (ii) increasing the operational temperature from 40 °C to 55 °C [22], and (iii) enhancing the specific surface area of the carrier material by using fragmented Siporax rings instead of intact elements [20].

Collectively, these adjustments resulted in a substantial improvement in CH_4_ formation rate (MFR), which increased from 0.4 to 13 L CH_4_ L^−1^ d^−1^, corresponding to an approximately fourfold enhancement relative to the stabilised baseline performance.

### 3.3. Performance of a 15 L Trickle-Bed Reactor

**Figure 2:**
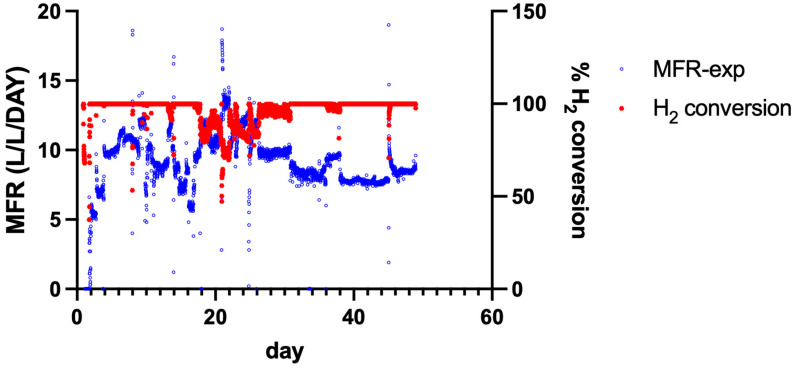
Methane Formation Rate and H_2_ conversion in a Trickle-Bed Reactor. The red line (right axis) represents the H_2_ conversion percentage, remaining close to 100% throughout most of the operational period. The blue data points (left axis) represent the experimentally determined CH_4_ formation rate (MFR), calculated from the CH_4_ fraction in the reactor off-gas. The sustained high H_2_ conversion in combination with the measured MFR demonstrates that the consumed hydrogen was quantitatively converted into CH_4._

The performance of a 15 L trickle-bed reactor (TBR) filled with Siporax™ was evaluated over a 45-day continuous operation period. Reactor performance was assessed based on the MFR and H_2_ conversion. Figure 2 shows the experimentally determined MFR, the modelled MFR, and the corresponding H_2_ conversion over time.

During reactor operation, periods were observed in which H_2_ and CO_2_ were detected in the reactor off-gas. The methane formation rate (MFR) was quantified from the measured CH_4_ concentration in the effluent gas. When total gas production exceeded the CH_4_ volume predicted by the stoichiometric model, the difference was interpreted as residual H_2_ and CO_2_. This non-methanized fraction was not included in the reported MFR. The experimental MFR values were compared with the modelled MFR, which was calculated from the inlet gas composition assuming stoichiometric conversion of H_2_ and CO_2_ according to:

$$MFR=\frac{input\;H_{2}(\frac{m^{3}}{day})}{4*V_{reactor}(m^{3})}$$

where

*H_2_-input* = volumetric H_2_ supply rate (m^3^ H_2_ d^−1^)
*V_reactor_* = working reactor volume (m^3^)
4 = stoichiometric conversion factor (4 mol H_2_ → 1 mol CH_4_)

Throughout the experiment, H_2_ conversion remained high, reaching 100% in most of the operational period (Figure 2). Temporal variability was observed in the experimentally determined MFR, particularly between days 10 and 30, whereas the modelled MFR remained relatively constant. Periods in which the experimentally calculated MFR exceeded the modelled MFR coincided with a reduction in the measured H_2_ conversion.

Periodic gas chromatography measurements confirmed the presence of H_2_ in the reactor off-gas during these periods. An overview of the MFR and H_2_ conversion trends is provided in Figure 2.

### 3.4. Gas Composition in the Trickle-Bed Reactor Performance over Time

**Figure 3:**
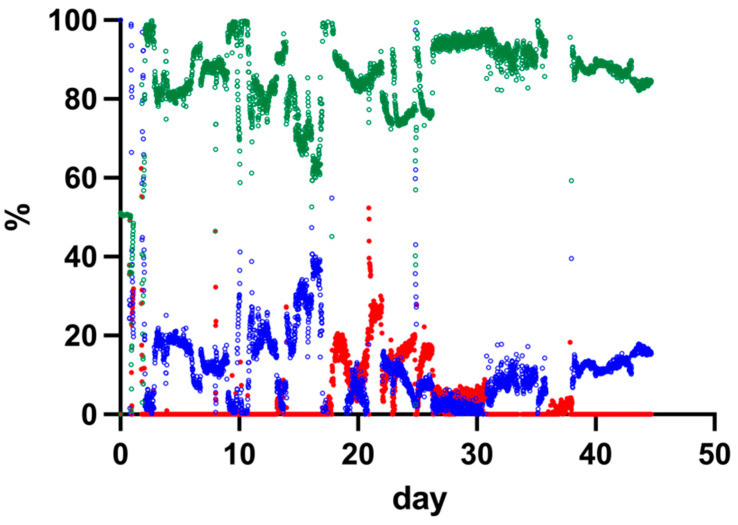
CH_4_, CO_2_, and H_2_ content in the off-gas of the trickle-bed reactor over time. % CH_4_: green, % CO_2_: blue; % H_2_: red.

Temporal changes in reactor off-gas composition were monitored during operation of the 15 L trickle-bed reactor. The concentrations of CH_4_, CO_2_, and H_2_ in the off-gas were determined over time. Figure 3 presents the measured gas composition profiles throughout the experimental period.

During reactor operation, fluctuations in gas composition were observed. After the initial start-up phase, CH_4_ concentrations stabilised, while H_2_ concentrations in the off-gas decreased to near-zero values after day 10. CO_2_ concentrations varied throughout the experiment.

Based on the stabilised operational period, the average CH_4_ concentration in the off-gas was calculated from day 26 to the end of the experiment and was 87.0 ± 4.0%. Variations in gas composition were observed between days 18 and 25, during which changes in the relative concentrations of CO_2_ and H_2_ occurred. The gas composition values represent relative volumetric percentages of the total measured off-gas.

### 3.5. Reactor Reactivation After Prolonged Inactivity

**Figure 4:**
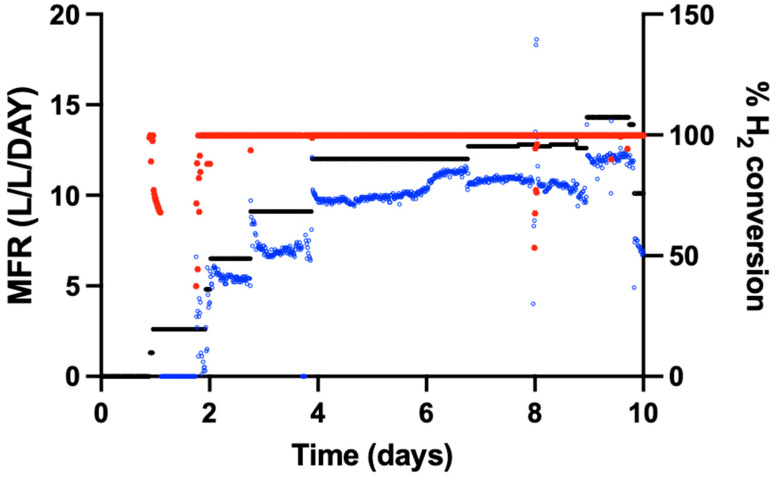
Start-up phase of the reactor after an 8-week storage period. The left *y*-axis represents the Methane Formation Rate (MFR, blue), and the right *y*-axis represents the H_2_ conversion (red) percentage. The *x*-axis indicates time. The black line shows the theoretical MFR based upon the H_2_ feed.

Following an extended idle period, the 15 L trickle-bed reactor was evaluated for reactivation performance. The reactor was stored at room temperature for 8 weeks after flushing with nitrogen gas. After the idle period, the reactor was reactivated by trickling with fresh inoculum and flushing with CO_2_. H_2_ was subsequently introduced. Once an H_2_ breakthrough is observed, the previous stable flow rate is considered the maximum biological conversion capacity. During the initial reactivation phase, minor H_2_ concentrations were detected in the off-gas. Within 24 h of H_2_ introduction, CH_4_ production resumed. The reactor reached CH_4_ formation rates in the range of approximately 10–12 L-CH_4_ L^−1^ reactor d^−1^ without additional mechanical or structural modification (Figure 4). CH_4_ production stabilised after this reactivation period .

### 3.6. Operation of the Trickle-Bed Reactor with Biogas as Feed Gas

The performance of the trickle-bed reactor was evaluated using biogas as the inlet gas. The biogas feed consisted of 58% CH_4_, 38% CO_2_, and approximately 4% N_2_, O_2_, and water vapour. The reactor performance was assessed based on MFR, methane evolution rate (MER), and off-gas composition.

Following the switch from pure gases to biogas, the CH_4_ concentration in the reactor off-gas remained approximately 80% despite an increased biogas feed rate. The CH_4_ evolution rate, expressed as L-CH_4_ L^−1^ reactor d^−1^, increased with increasing biogas supply and represents the total CH_4_ flux through the reactor column.

**Figure 5:**
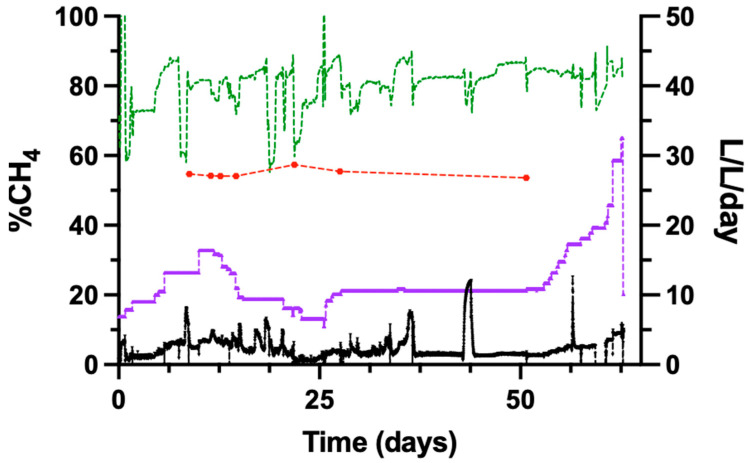
Biogas’s CH_4_ concentration (left axis) and MFR and MER (right axis) over 52 days in percentage and l/l/day, respectively. The green dashed line represents the CH_4_ concentration in the off-gas leaving the reactor, while the red line shows the measured CH_4_ percentage in the biogas entering the reactor (input). The black line represents the Methane Formation Rate (MFR), which indicates the rate of CH_4_ production, and the purple line represents the Methane Evolution Rate (MER), which combines the CH_4_ in the biogas with newly formed CH_4_ in the reactor.

The MFR, defined as the volume of newly produced CH_4_ produced from the conversion of CO_2_ and H_2_, was lower during biogas operation than during experiments using pure CO_2_ and H_2_ as substrates. Figure 5 shows the temporal evolution of CH_4_ content in the biogas feed and the upgraded off-gas. Measurements of the biogas inlet composition, obtained from a bypass of the feed stream, showed an average CH_4_ content of 54.8 ± 1.2%. The average CH_4_ concentration in the upgraded off-gas during the experimental period was 79.4 ± 12.9%.

Fluctuations in off-gas CH_4_ concentration were observed throughout the 52-day experiment.

### 3.7. Comparison Between Experimental and Theoretical Methane Formation Rates

**Figure 6:**
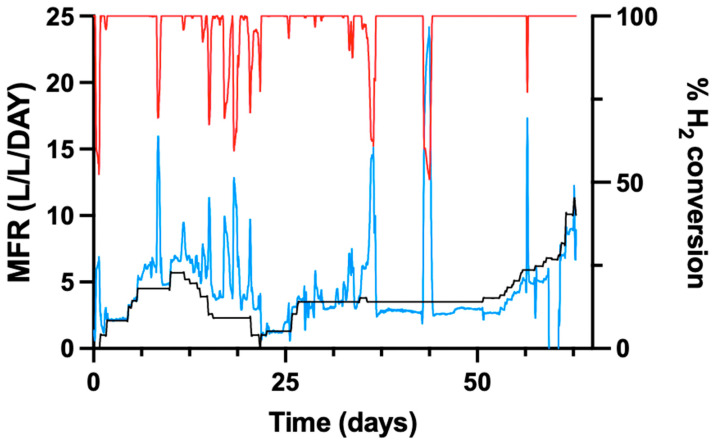
Comparison of theoretical and experimental methane formation rates (MFR), with data smoothed using a 12-neighbour average. The black line represents the theoretical MFR. It is important to note that fluctuations in the theoretical MFR were caused by deliberate interventions to stabilise the reactor and varying input flows. The blue line depicts the experimental MFR over time, with fluctuations and spikes reflecting variability in the experimental process. The red line illustrates the H_2_ conversion.

The experimentally determined MFR-exp was compared with the theoretical MFR-model calculated from the inlet gas composition. Figure 6 presents the temporal evolution of both MFR values during reactor operation.

Throughout the experimental period, the experimentally determined methane formation rate (MFR-exp) fluctuated despite stable inlet gas flow rates. These deviations likely reflect transient mass transfer limitations and dynamic biofilm activity rather than changes in substrate supply.

H_2_ conversion showed temporal variation during reactor operation. During the initial phase of operation, H_2_ conversion values approached 100%. Subsequently, periods of reduced H_2_ conversion were observed, followed by a later phase in which H_2_ conversion and MFR-exp increased and became more stable. Around day 30, both H_2_ conversion and MFR-exp showed a general upward trend.

An overview of the experimental and theoretical MFR values and the corresponding H_2_ conversion is shown in Figure 6.

### 3.8. Gas Residence Time During Operation of the Trickle-Bed Reactor

The gas residence time (GRT) in the trickle-bed reactor was calculated under different gas feed compositions. When operating with pure H_2_ and CO_2_, the calculated GRT was 101.1 min at an MFR of 10 L-CH_4_ L^−1^ reactor d^−1^. Under biogas operation, where CH_4_ was present in the inlet gas, the calculated GRT decreased to 52.3 min at the same MFR.

The reduction in GRT reflects the increased total gas flow rate due to CH_4_ in the inlet gas. Calculated GRT values were used to compare reactor operation under different feed gas compositions.

## 4. Discussion

A trickle-bed reactor provides a favourable configuration for ex situ biomethanation by combining counter-current gas–liquid flow with immobilised biomass on a fixed bed. This configuration enables high biomass retention and enhanced gas–liquid interfacial area, both of which are critical for hydrogenotrophic methanogenesis. However, reactor performance is strongly influenced by the interaction between carrier material properties, hydrodynamics, and operational conditions [23].

In this study, Siporax™ was selected as a carrier material based on its exceptionally high specific surface area and three-dimensional pore structure [20]. Compared to commonly used carrier materials reported in the literature, Siporax™ provides almost two orders of magnitude higher surface area per unit volume, which is expected to support extensive microbial attachment and biofilm development [24]. The characterisation experiments demonstrated that both whole and fragmented Siporax rings exhibit high surface-area densities (270–382 m^2^ L^−1^), confirming their suitability as biofilm carriers in TBR systems.

At the same time, the results highlight that increased surface area is accompanied by trade-offs related to packing behaviour and hydrodynamics. Fragmentation of Siporax rings resulted in a higher measured void volume compared to whole rings, despite the smaller particle size. This counterintuitive effect can be attributed to irregular particle shape and less efficient packing of the carrier material, which increases the open space between particles. These differences in void volume directly affect the distribution of gas flow and gas residence time, both of which are critical parameters in H_2_-driven biomethanation.

The area-to-void-volume ratio provides a useful metric for balancing biofilm support and gas–liquid interactions. Although whole and fragmented rings exhibited similar ratios, the more complex carrier-material arrangement in fragmented Siporax is expected to promote greater turbulence and local mixing. Such effects can enhance gas–liquid mass transfer, particularly when H_2_ availability limits methane formation. However, increased turbulence and surface area may also increase the risk of excessive biofilm growth, clogging, or uneven flow distribution, emphasising the need for careful operational control [16].

Gas residence time emerged as a key factor linking carrier material choice to reactor performance [25]. Calculated gas residence times differed substantially between whole and fragmented rings and were further reduced during biogas operation due to the presence of CH_4_ in the inlet gas. While CH_4_ does not participate in the methanation reaction, it increases total gas flow and thereby reduces the effective residence time available for CO_2_ and H_2_ conversion [26]. This effect explains the observed reduction in methane formation rate during biogas operation compared to experiments using pure CO_2_ and H_2_, despite maintaining high H_2_ conversion.

The use of a practical H_2_:CO_2_ molar ratio of 3.7:1, rather than the stoichiometric 4:1, further illustrates the interaction between microbial activity and reactor design. This deviation accounts for CO_2_ assimilation into microbial biomass and reflects realistic operating conditions in biological systems. Under these conditions, effective gas residence time increases substantially, partially compensating for the dilution effects introduced by CH_4_ in the feed gas. Consequently, H_2_ and CO_2_ are supplied at a lower flux relative to the available biofilm surface, partially mitigating dilution effects caused by CH_4_ in the feed gas [27].

Overall, the results demonstrate that the selection of carrier material in TBRs cannot be optimised solely on the basis of surface area. Instead, reactor performance is governed by a balance between surface area, void volume, gas residence time, and flow distribution. Siporax™ proved to be an effective carrier material for high-rate biomethanation. In practice, the use of Siporax™ in trickle-bed biomethanation reactors requires tight control of gas flow, gas recirculation, and liquid distribution to ensure sufficient gas–biofilm contact while avoiding channelling, excessive pressure drop, and biofilm instability [14,28,29]. These findings underline the importance of integrating carrier material properties with reactor design and process control strategies when scaling up TBR technology for biogas upgrading and power-to-gas applications [30]. From an economic perspective, ex situ biomethanation is primarily constrained by H_2_ supply costs, with electricity price and electrolyser CAPEX dominating total system costs [14]. The bioreactor affects economics indirectly by determining volumetric CH_4_ productivity (MFR) and, in turn, the required reactor volume. Conventional wet reactors, such as CSTRs or UASB-type systems, benefit from technological maturity but are inherently limited by H_2_ gas–liquid mass transfer, resulting in large reactor volumes at high H_2_ loading rates. Reactor concepts that enhance the gas–liquid interfacial area, such as trickle-bed reactors, are therefore frequently considered economically promising, as they enable more compact designs with relatively low auxiliary energy demand [31]. A detailed techno-economic analysis is outside the scope of this study; the results are intended to position the reactor concept within qualitative economic trade-offs.

## 5. Conclusions

This study demonstrates that thermophilic trickle-bed reactors (TBRs) can achieve high-rate ex situ biomethanation under laboratory-scale conditions . Methane formation rates were consistently high, with CO_2_ conversion approaching 90% and H_2_ conversion exceeding 99% during stable operation using pure CO_2_ and H_2_ as feed gases. These results confirm the suitability of TBR configurations for hydrogenotrophic biomethanation, provided that sufficient H_2_ is supplied relative to CO_2_.

The experimental results indicate that CO_2_ conversion is strongly constrained by H_2_ availability and reactor operating conditions. While near-complete H_2_ conversion was achieved, CO_2_ conversion remained below full stoichiometric conversion, highlighting the importance of H_2_ supply control and gas–liquid mass transfer in determining overall reactor performance.

The use of wastewater as a trickling medium enabled stable reactor operation while simplifying nutrient supply, although it introduced variability that may affect reproducibility. Despite this variability, the reactor maintained stable performance during extended operation and recovered rapidly after prolonged inactivity, indicating a high degree of operational robustness.

Overall, the results show that trickle-bed reactors represent a promising platform for high-rate biomethanation and biogas upgrading. Further work is required to evaluate long-term stability and performance under biogas feed conditions and to assess scalability under industrially relevant operating regimes.

## Figures and Tables

**Table 1:** Characteristics of Siporax^TM^.

Parameter	Siporax	¼ Siporax	Unit
Total Area	270	382	m^2^/L
Gas-Filled Volume (VOS)	314	457	mL/L
Area-Void Volume Ratio	859	835	m^2^/L
GRT (N_2_, 10 mL/min)	31	46	min
GRT (H_2_/CO_2_ 10 mL/min; ratio 3.7)	147	214	min

## Data Availability

The data supporting the findings of this study are not publicly available due to internal project agreements but may be shared by the corresponding author upon reasonable request.

## Supplementary Materials

The following are available online at https://www.mdpi.com/article/10.3390/bioengineering13040382/s1.

## Abbreviations

The following abbreviations are used in this manuscript:

AD	Anaerobic Digestion
AFEX	Ammonia Fibre Expansion
BD	Biodegradability
BMP	Biochemical Methane Potential
CH_4_	Methane
CO_2_	Carbon Dioxide
CSTR	Continuous Stirred Tank Reactor
GRT	Gas Retention Time
H_2_	Hydrogen
LC	Lignocellulose
MFR	Methane Formation Rate
MER	Methane Evolution Rate
NBUS	Natural Biomass Utilisation System
NH_3_	Ammonia
OLR	Organic Loading Rate
RNG	Renewable Natural Gas
SMP	Specific Methane Production
SRT	Solid Retention Time
TBR	Trickle-Bed Reactor
TBP	Theoretical Biogas Potential
VFAs	Volatile Fatty Acids
VS	Volatile Solids
