# Supplementary material for: An Anaerobic Trickle-Bed Reactor Filled with Siporax™ as a Novel Approach for Biomethanation of Hydrogen and Carbon Dioxide
Source: Bioengineering (Basel). 2026 Mar 26;13(4):382. doi: 10.3390/bioengineering13040382 (PMC13113737; doi:10.3390/bioengineering13040382)
Supplement: Supplementary file 1 [file bioengineering-13-00382-s001.zip › bioengineering-4115373-supplementary.pdf]

Supplementary Information – Gas Residence Time Calculations

Gas residence time (GRT)

The Gas Residence Time (GRT) was calculated as: GRT = V_{gas} / Q_{gas}, where Vgas is the gas-filled volume (mL), equal to the volume of open space (VOS), and Qgas is the inlet gas flow rate (mL min^{-1}).

Gas-filled volume (Vgas)

$V_{gas} = V_{reactor} – V_{packing}$

Correction for gas contraction during biomethanation

Hydrogenotrophic methanation follows: $CO_2 + 4H_2 \rightarrow CH_4 + 2H_2O$. In practice, a molar ratio of H_2/ CO_2 = 3.7:1 was applied, with hydrogen assumed to be the limiting reactant. The total molar inlet flow is therefore 3.7 + 1 = 4.7. Assuming complete hydrogen conversion, this results in volumetric contraction of the gas phase by a factor of 4.7.

Equivalent gas residence time

The equivalent gas residence time was calculated as: $GRT_{eq} = GRT \times 4.7$
